# Liquid–Liquid Phase Separation in Crowded Environments

**DOI:** 10.3390/ijms21165908

**Published:** 2020-08-17

**Authors:** Alain A. M. André, Evan Spruijt

**Affiliations:** Institute for Molecules and Materials, Radboud University Nijmegen, Heyendaalseweg 135, 6525 AJ Nijmegen, The Netherlands; alain.andre@ru.nl

**Keywords:** liquid–liquid phase separation, intrinsically disordered proteins, crowding, membraneless organelles

## Abstract

Biomolecular condensates play a key role in organizing cellular fluids such as the cytoplasm and nucleoplasm. Most of these non-membranous organelles show liquid-like properties both in cells and when studied in vitro through liquid–liquid phase separation (LLPS) of purified proteins. In general, LLPS of proteins is known to be sensitive to variations in pH, temperature and ionic strength, but the role of crowding remains underappreciated. Several decades of research have shown that macromolecular crowding can have profound effects on protein interactions, folding and aggregation, and it must, by extension, also impact LLPS. However, the precise role of crowding in LLPS is far from trivial, as most condensate components have a disordered nature and exhibit multiple weak attractive interactions. Here, we discuss which factors determine the scope of LLPS in crowded environments, and we review the evidence for the impact of macromolecular crowding on phase boundaries, partitioning behavior and condensate properties. Based on a comparison of both in vivo and in vitro LLPS studies, we propose that phase separation in cells does not solely rely on attractive interactions, but shows important similarities to segregative phase separation.

## 1. Introduction

The cytosol is a complex mixture of macromolecules, including proteins, nucleic acids and polysaccharides. Although the individual concentrations of these macromolecules varies between cell types and organisms, their combined concentration can reach up to 400 mg·mL^−1^ [1,2]. This high concentration means that the cytosol is densely packed with macromolecules. Under these conditions, biochemical processes are strongly affected by limited diffusion and volume exclusion [3,4].

Traditionally, biochemists have sought to understand biochemical processes by studying them in isolation, using minimal models in the form of purified proteins. These in vitro (in this review used to indicate cell-free) studies were commonly conducted in aqueous buffer solutions, where the effect of crowding was omitted. To study the effect of crowding in vitro, scientists started to use synthetic polymeric molecules such as polyethylene glycol (PEG), Ficoll and dextran [5]. In addition to synthetic polymers, globular proteins such as bovine serum albumin (BSA), have been used a step towards more biologically relevant crowders [6]. However, the cytosol also contains high levels of disordered proteins and RNAs that are not specifically neutral [7]. Some of them form macroscopic puncta (or bodies/foci/granules) in the cell, creating an additional level of complexity.

**Figure 1:**
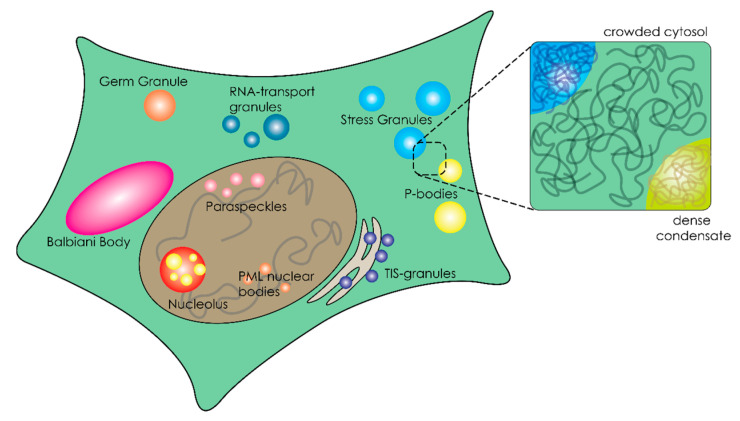
Schematic overview of a eukaryotic cell containing membranous and membraneless organelles.

Recent developments in cell biology aimed at understanding the cellular organization have shed new light on the physical state of cellular fluids. Instead of a crowded, single-phase mixture of macromolecules, the cytoplasm and nucleoplasm of many cells appear dotted with condensates (granules, bodies, foci and puncta) that are separated from the surrounding fluid (Figure 1) [7]. These condensates seem to be dynamically formed and dissolved over time [8]. Many of these micro-niches in the cell have liquid-like properties, such as nucleoli, germ granules, stress granules and paraspeckles [9], and they have been collectively described as membraneless organelles (MLOs) [10,11] or biomolecular condensates [9]. Many MLOs are likely formed through liquid–liquid phase separation, resulting in very dense droplets [9,11].

To elucidate the effect of crowding on MLOs, in vitro models using purified proteins combined with synthetic crowding agents have been employed, similar to the first studies of the effect of crowding on enzyme activity. In this review, we discuss the possible effects of crowded environments on phase separation. To understand this, we first introduce the theoretical framework that has been used extensively to explain crowding effects on biochemical processes, and highlight the factors that are particularly relevant to phase separating proteins. We then briefly describe the characteristics of micro-compartments formed by phase separation. In the remainder of this review, we give an overview of the use of crowders in in vitro MLO studies, and the observed effects of crowding on phase separation, partitioning and the biophysical properties of condensates.

## 2. The Effect of Macromolecular Crowding on Biochemical Processes

Macromolecules occupy as much as 30% of the cellular volume [2], which makes the intracellular space a crowded place. The abundance of macromolecules influences all aspects of biomolecular behavior, including protein folding, complexation, aggregation, and enzymatic activity. Since the importance of crowding was recognized in the 1960s, the effects of macromolecular crowding on biomolecules have been extensively studied in a cell-free setting. For a comprehensive overview, we refer to several excellent reviews by Ellis [3,12,13,14], Minton [4,15,16,17], and Rivas [5,18] on the physical aspects of crowding, and Zhou [19,20,21] on protein folding. To understand the role of crowding in liquid–liquid phase separation (LLPS), it is essential to outline some key concepts and approaches in crowding research. Therefore, we will briefly describe the principles of excluded volume and depletion here, and discuss their effects on biomolecular structure and assembly, followed by additional effects of crowding on biomolecular dynamics and reactions, and finally, we discuss the most common experimental approaches to mimic cellular crowding.

### 2.1. Excluded Volume Theory

**Figure 2:**
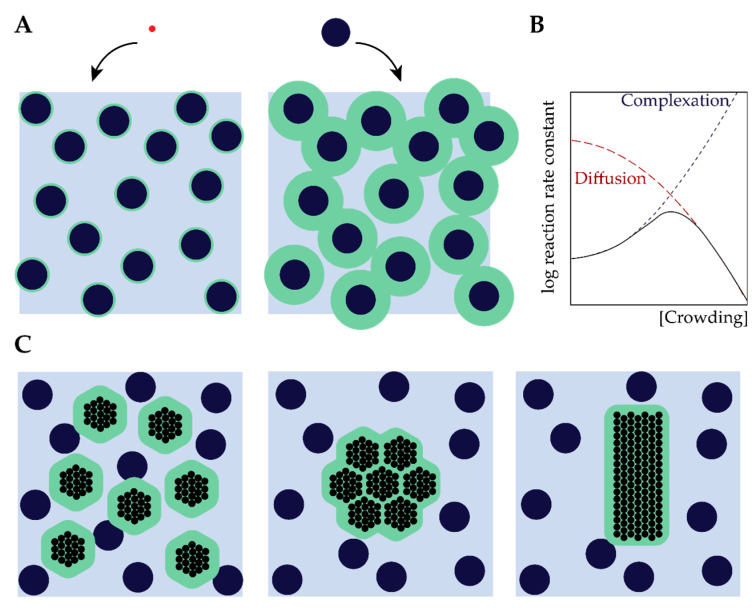
Excluded volume effect of crowding. (**A**) A small particle (red) experiences a smaller excluded volume than a large particle (black sphere). The blue area represents the free space for the particle; for a large particle the effective free volume is limited. (**B**) Many biochemical processes display a maximum rate at some optimum crowding, as crowding enhances complexation (blue curve), but it also reduces diffusivity (red curve) (**C**) The effect of depletion forces, bringing molecules together reduces the total excluded volume. (Figure 2A is adapted from Minton 2001 [23], Figure 2B is adapted from Ellis 2001 [3], and Figure 2C is adapted from Richter 2008 [24]).

Central to the effects of macromolecular crowding on biomolecular structure and dynamics is the notion that different species cannot occupy the same space: they exclude other species from a certain volume [3,12,16,22]. The excluded volume is directly related to the size of molecules: large molecules (such as proteins) experience much larger excluded volumes than small molecules (Figure 2A). More generally, the excluded volume depends on the size but also the shape of both the biomolecules of interest and the crowders [3]. An important consequence of this excluded volume is that the effective concentration of these biomolecules can rise by several orders of magnitude compared to the global concentration, which can have a strong effect on the rates of biochemical processes (Figure 2B) [23].

Rearranging of biomolecules can also result in overlapping of excluded volumes (Figure 2A) and, hence, a net increase in the volume that is available for crowders [23]. For inert crowders such an increase in available volume is entropically favorable, and manifests itself as an attraction between the biomolecules, which is known in colloid–polymer mixtures as the depletion attraction [24,25]. It is important to emphasize that the attraction depends strongly on the actual intermolecular interactions between the biomolecules of interest and the crowders (depletant): the simplified Asakura–Oosawa–Verwey model that is often used to quantify depletion interactions only holds for inert (hard-sphere) depletants. If the crowders are weakly attracted to the biomolecules, the depletion attraction diminishes rapidly and may disappear completely [26], a case we discuss in more detail below in Section 2.4. Soft repulsion, on the other hand, may enhance the depletion attraction [27].

### 2.2. The Effect of Crowding on Biomolecular Structure and Assembly

As illustrated in Figure 2C, the general consequence of crowding-induced depletion is that compact states of biomolecules are favored: protein folding, oligomerization, complexation and aggregation are all more favored in crowded environments compared to the non-complexed state [24,25]. Since LLPS involves a condensation of proteins, it seems logical to assume that crowding favors phase separation for precisely the same reasons. However, the biomolecules involved in LLPS are usually different from the globular proteins (commonly simplified as spherical objects) that are typically considered in crowding studies. Proteins that play an important role in LLPS are often intrinsically disordered, and the effect of crowding on such disordered biomolecules is far from trivial. A detailed account of the role of crowding on disordered proteins was recently written by Fonin et al. [28]. Here, we highlight two cases that are of particular relevance for LLPS.

First, intrinsically disordered proteins or regions of proteins (IDPs/IDRs) can become more compact under crowded conditions, which could lead to secondary structure formation of so-called foldons [19,28,29,30,31], for example, histone protein 1 (H1), a protein that is involved in chromatin regulation and has recently gained attention as condensate regulator through the C-terminal IDR [32,33]. In the case of H1, the C-terminal domain (CTD) was shown to gain secondary structure in the form of a molten globular domain under polyethylene glycol (PEG) and Ficoll-70 conditions [34]. Furthermore, secondary structure formation, including α-helix and β-turns, was also observed upon binding of DNA [35], indicating the CTD of H1 acts as a semi-foldon. However, when studied in LLPS, extensive NMR analysis by Stott and co-workers showed that the CTD remains unstructured even upon complexation with DNA in a crowded condensed form [32].

Second, crowding promotes intermolecular interactions of proteins, which can lead, for example, to protein aggregation [36,37,38]. For example, synuclein shows enhanced aggregation under crowded conditions [37,38]. This process is relevant to phase separation as many proteins such as fused in sarcoma (FUS) and heterogenous nuclear ribonucleoprotein A1 (hnRNPA1) have shown a transition from a dynamic fluid state to a solid aggregate state [39,40]. These solidification transitions have been linked to neurodegenerative diseases such as Alzheimer’s disease. In Section 4.3 we will discuss some examples where crowding changes the material properties of condensates in vitro.

### 2.3. From Assembly to Reactions

Crowding not only affects the structure and assembly of biomolecules, it also influences the rates of biochemical reactions [3,22]. It is commonly accepted that increasing crowdedness initially leads to enhanced rates of biochemical processes that involve binding or complexation [4,22]. A combination of higher effective concentrations (see Section 2.1) and a shift in chemical equilibria towards the energetically favored compact, complex states (see Section 2.2) results in higher predicted rates of many enzymatic processes. Indeed, crowding has been found to accelerate processes such as gene expression [41], DNA replication [42] and protein phosphorylation [43]. Moreover, by favoring bound states, crowding also affects processivity and stochasticity of processes [43,44]. The opposite effect, in which crowding decreases the activity of certain enzymes, has also been reported, and is generally attributed to a reduced conformational flexibility of enzymes that require significant changes in conformation for their activity [45].

A further increase in crowding typically results in decreasing rates of many biochemical processes, which implies that the rate goes through a maximum at an intermediate crowder concentration (Figure 2B) [3,22,23]. The decrease in rate is a result of strongly reduced diffusivities of the biomolecules involved in a reaction at high crowding [3,46]. The corresponding maximum has been observed experimentally for cell-free gene expression in solution [47]. It is interesting to note that an enhanced enzyme activity, similar to what is commonly seen in intermediately crowded conditions, has also been proposed to occur for “overcrowded” condensates [48,49,50]. Whether this is a consequence of crowding, or has a different origin, is still an open question, which is beyond the scope of this review.

### 2.4. Mimicking Cellular Crowding

To investigate the role of crowding in biochemical reactions, assembly processes, and LLPS, in vitro experiments are generally performed using model crowding agents at varying concentrations. General considerations for selecting an appropriate crowding agent are: (i) the crowding agent should not interact specifically with the biological system [5,51], and (ii) the crowding agent should be highly water soluble [5,16]. As we mentioned earlier, roughly 30% of the cellular volume is occupied by macromolecules. Therefore, these crowding agents should reach concentrations up to 300 mg·mL^−1^ without precipitation [2,51]. The combination of these two criteria means that multiple crowding agents should be tested to ensure that observed effects are not specific [5].

Polymers such as PEG and Ficoll are widely used as model crowding agents. These polymers might be relatively inert in most systems, but they are very different from most cellular components. As a step towards cellular crowding, highly water-soluble proteins have been introduced, such as BSA and lysozyme [19,46,52]. Considering the first criterium of the crowder being an inert molecule, proteins increase this complexity due to the large variety of amino acids that could introduce weak non-covalent interactions. Furthermore, an advantage of polymeric crowders is that they are usually available in a the large variety of sizes (e.g., PEG can range from 2.5 kDa up to 20 kDa), allowing systematically varying the size of the crowders, while proteins have a fixed size (e.g., BSA is 68 kDa and lysozyme 14 kDa) [5,52].

Although these synthetic crowders have given important insights into the thermodynamic effects of ideal crowding, it is questionable how well Ficoll, dextran or PEG can mimic the cytosol. To make this point, Elcock wrote in 2010: ‘I never once came across one [paper] that began: Ficoll and dextran are incredibly important molecules and it is therefore vital that we understand their effects on protein folding and protein-protein associations’ [53]. Therefore, bottom-up approaches have recently sought to better understand the role of cellular crowding by using purified and concentrated lysates as natural crowding solutions. For example, Huck and co-workers managed to condense *Escherichia coli* lysate, and found enhanced transcription and translation rates compared to the dilute phase [41]. Furthermore, at a single protein level, lysate showed non-specific interactions that inhibited folding of a FRET-based crowding sensor, while the same sensor did show enhanced folding in Ficoll and PEG-rich media [26]. The usage of dense lysate thus narrows the gap between complicated in cell (in vivo) experiments (e.g., in cell NMR and super-resolution microscopy) and in vitro experiments using only polymers.

Lysate might sound like the best replacement of polymeric crowders, but it should not be thought of as the perfect mimic of the real, complex cellular milieu, as Rivas and Minton have pointed out [18]. First, because lysates have been cleared of lipid components, membrane–tracer interactions are not present. Second, lysate contains a large amount of uncharacterized proteins which might interact either specifically or non-specifically with probe molecules. Third, cells are known to contain microcompartments, and the preparation of lysate could distort the stability or formation of these compartments. In short, the ideal model crowded environment for systematic in vitro studies does not exist yet, and to improve our understanding of the role of crowding on structure, dynamics and phase behavior of biomolecules, a combination of approaches is crucial.

## 3. Organization of the Crowded Cytosol through Overcrowded Condensates

Cells are not only crowded, they are also compartmentalized. Biomolecular condensates, or membraneless organelles, are an important class of microcompartments that function as cellular regulators [51]. These microcompartments stand out from the traditional subcellular compartments, such as mitochondria and lysosomes, because they lack a surrounding membrane [9]. Their separate role in cellular organization was first recognized for P-granules [8], but the number of examples grew rapidly and include stress granules [54,55], paraspeckles [56], P-bodies [57], nuage bodies [58] and nucleoli [59,60]. These compartments form by condensation of proteins, nucleic acids and enzymes into dense droplets, which have liquid properties [7,8,59]. To understand the effect of crowding on phase separation, it is important to first highlight some of the typical structural features of the biomolecules involved in phase separation.

### 3.1. Liquid–Liquid Phase Separation of Proteins and Nucleic Acids within Cells

**Figure 3:**
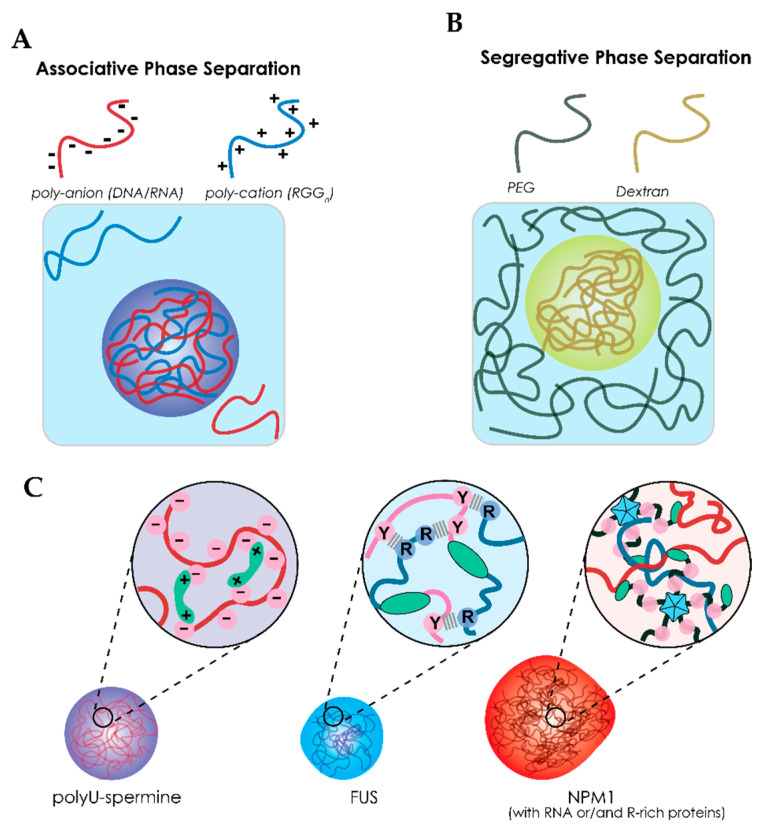
Liquid–liquid phase separation of proteins and polymers. Illustration of the difference between (**A**) associative phase separation which relies on attration between two macromolecules and (**B**) segregative phase separation which relies on repulsive interactions. (**C**) The three model systems from Section 4 which contains poly-U–spermine; FUS; NPM1 condensates. Poly-U–spermine condensates rely solely on charge interactions between RNA (negative) and spermine (positive). Increasing complexity, FUS has cation–π interactions between arginine (RGG motif, cation) and tyrosine ([G/S]Y[G/S] domain, π) residues within the disordered domains. RNA could play a role but is not included in the crowded studies discussed in Section 4. Finally, NPM1 has an oligomerization domain and a nuclear-binding domain in addition to charge interactions.

The formation of biomolecular condensates has been linked to liquid–liquid phase separation (LLPS), a process where macromolecules (e.g., DNA, RNA, and proteins) associate with each other through weak non-covalent interactions to form dense liquid droplets, as illustrated in Figure 3A [9,11]. Proteins that are enriched in these droplets tend to have extensive intrinsically disordered regions (IDRs) [61,62,63]. Harmon et al. describes phase separation of IDR-containing proteins using a model of stickers and spacers [64]. This model includes some important features to describe the liquid behavior of condensates, namely: weak non-covalent interactions (stickers), the valency (number of interactions) and flexibility (IDRs that do not interact) [64,65]. Although Harmon et al. describe the stickers as single amino acids or short linear amino acids motifs [64], these motifs are not the only factors that can drive phase separation. Other examples of interaction domains include structural binding domains and oligomerization domains, both of which are widely present in the phase-separating proteome [9,11].

A variety of motifs in low-complexity domains (LCDs) have been identified as weak binders. Such motifs include arginine-rich motifs (RGG) as found in FUS [66,67], Ddx4 [58,61], and LAF1 [68,69], prion-like domains (PrLD) containing repeats [G/S]Y[G/N/A/S]AQ as found in FUS [39,67,70,71]. The RGG and PrLD domains can form cation–π interactions. These are the most important interactions of FUS and will be further discussed in Section 4 (Figure 3C).

Structural binding domains can be found in the form of RNA-recognition motifs (RRM), zinc fingers (ZnF) and nucleic acid binding domains (NBD), which all interact with RNA and can increase certain sequence specificity [67,72]. Structural domains are not limited to RNA binding domains; some also involve protein–protein interactions, for example, through PRM binding domains and SH2 domains [65,73]. Finally, oligomerization domains are important for increasing the number of binding sites of a protein. G3BP1 is known to form dimers, doubling the number of binding sides [74]. A more remarkable example is nucleophosmin (NPM1) which forms pentamers. This five-fold increase is important for phase separation with rRNA as it relies on a single NBD (Figure 3C) [75].

### 3.2. The Role of RNA in Liquid–Liquid Phase Separation

In addition to proteins, many biomolecular condensates are also enriched with RNAs. Although most condensates that are enriched in RNA contain RNA binding proteins [63,76,77], there is strong evidence that RNA–RNA interactions contribute to phase separation as well [78]. The lab of Gladfelter has shown that RNA structure dictates the formation of distinct Whi3 condensates both in vivo and in vitro [79,80]. The importance of RNA within biomolecular condensates has recently been reviewed by Roden and Gladfelter [81].

### 3.3. Biophysical Properties of Membraneless Organelles

As a consequence of phase separation, the interior of these membraneless compartments is even more crowded than the cytoplasm. It has been proposed that this overcrowding could play a role in several suggested functions of MLOs, such as activation (e.g., nucleation of fibrils) and changing reaction kinetics (e.g., bringing reactants in close proximity) [9,50,82,83]. Another consequence of overcrowding is the reduced water content of condensates, which can affect other noncovalent interactions involved, such as base pairing and host–guest binding [84]. A recent example was given in the case of the DEAD box helicase Ddx4, which forms condensates through simple phase separation [58,61,85]. Due to the relatively hydrophobic nature of the amino acids of Ddx4, the interior of Ddx4 condensates is more similar to organic solvents, such as DMSO, than to water [85]. These condensates are able to “filter” ssDNA from a pool of both dsDNA and ssDNA by preferential uptake [58,85]. Thus, biophysical properties of condensates could affect sequestration of molecules and reaction selectivity [86].

To determine the molecular mechanisms underlying MLO formation and functioning, in vitro studies are indispensable and have already provided tremendous insight into the characteristics and potential roles of MLOs on biomolecular processes [9,11,50,76,82,83,86,87,88,89,90,91,92,93,94]. In these in vitro experiments, the effects of pH, temperature and ionic strength on phase behavior are generally explored. However, the effect of macromolecular crowding on LLPS is usually not discussed in great detail, while crowding agents are commonly added to in vitro LLPS models. It is therefore pertinent to look into the relation between macromolecular crowding and LLPS to understand the relevance of these studies for LLPS in living cells.

## 4. How does Crowding Affect Liquid–Liquid Phase Separation?

Macromolecular crowding agents such as PEG, Ficoll and dextran are commonly used in cell-free LLPS studies, as summarized in Table 1. Some of the earliest reported in vitro phase separated condensates, such as the Nck/N-WASP [65], Ddx4 [58], and LAF-1 [69] were formed in non-crowded buffers. However, as research into phase separation of cytoplasmic proteins progressed, the use of macromolecular crowders became more ubiquitous. Currently, crowding agents are added in almost all studies of in vitro model MLOs (Table 1). As an example, Tau [95,96,97], G3BP1 [97,98], hnRNPA1/3 [40,68,99,100], EFhd2 [97], and TAF15 [99] are generally studied in the presence of 10% (polymeric) crowders. These polymeric crowders vary in molecular weight: commonly used Ficoll-70 has an average molecular weight (*M*_w_) of 70 kDa; PEG is used in a range of 3.35 kDa up to 20 kDa. It is important to consider the *M*_w_ of crowders as these can have different effects on phase separation, as shown for lysozyme [101].

In studies of protein condensates from the nucleoplasm, crowding agents are also commonly added. To study phase separation of the polymerase and mediator complex, Guo and co-workers used 16% Ficoll-400 [102], a much larger crowder than used in studies on cytosolic stress granule components, where Ficoll-70 is typically used. Proteins involved in nucleolus formation, nucleophosmin (NPM1) and fibrillarin (FBL/Fib1), have been exposed to three types of crowders as well in varying concentrations, ranging from no crowding to 10–15% crowding [103,104,105]. Although the degree of crowding in the nucleoplasm is considered to be higher than in the cytoplasm, so far in vitro studies make no clear distinction between the two with respect to the size or concentration of crowding agents added.

After analyzing the studies summarized in Table 1 for the use of crowding agents, we noticed that systematic studies on the effect of crowding remain scarce. Nonetheless, we can distinguish at least three main effects caused by crowding: (i) macromolecular crowding can induce LLPS (Section 4.1); (ii) macromolecules may co-condense or partition into condensates (Section 4.2); (iii) macromolecular crowders can change the biophysical properties of condensates (Section 4.3).

### 4.1. Crowding-Induced Phase Separation

Not many studies on biomolecular phase separation systematically analyze the influence of crowding; even the simple presence or absence of crowding agents in buffers used is not always explicitly stated in papers. However, there is ample evidence that crowding does affect LLPS. Two examples that highlight the importance of crowders for the formation of condensates are: (i) for TIA-1, a stress granule protein, there were no clear condensates detected in the absence of crowding. The addition of 10% PEG was crucial to detect condensates by microscopy. (ii) FCA, a floral repressor protein, showed very small dots without PEG, but the addition of 10% PEG increased the dots to tens of microns, sizes that are similar to what is observed in other in vitro phase separation experiments. 

In most cases, the addition of crowding agents leads to a decrease in the critical protein concentration required for phase separation, or it is essential to observe phase separation at all (Table 1). Crowding thus appears to induce phase separation in mixtures that would not show phase separation without crowders. In the case of hnRNPA1 and FUS, for instance, crowding was found to alter the phase diagram towards condensate formation at a lower protein concentration. Lin and co-workers showed that at 10% BSA, the critical concentration of hnRNPA1 required for phase separation shifted from the micromolar regime to nanomolar regime [100]. Similarly, Kaur observed a minimal FUS concentration of 0.5 µM when exposed to 17.5% PEG-8k [106]. More recently, Yang and co-workers showed that doubling the Ficoll concentration to 10%, caused a 4-fold reduction in the critical G3BP1 concentration [97]. These results show a strong correlation between both polymeric and protein crowders and the critical protein concentration for phase separation. While it has been suggested that the response of IDRs to crowding is very heterogeneous, because IDRs could either (partially) fold or unfold depending on the amount type of crowding agent [28], the examples reported in literature (Table 1) mostly suggest that crowding enhances phase separation. It is possible that this correlation is biased, however, as the diversity of crowding agents used is limited to PEG, Ficoll and dextran.

**Figure 4:**
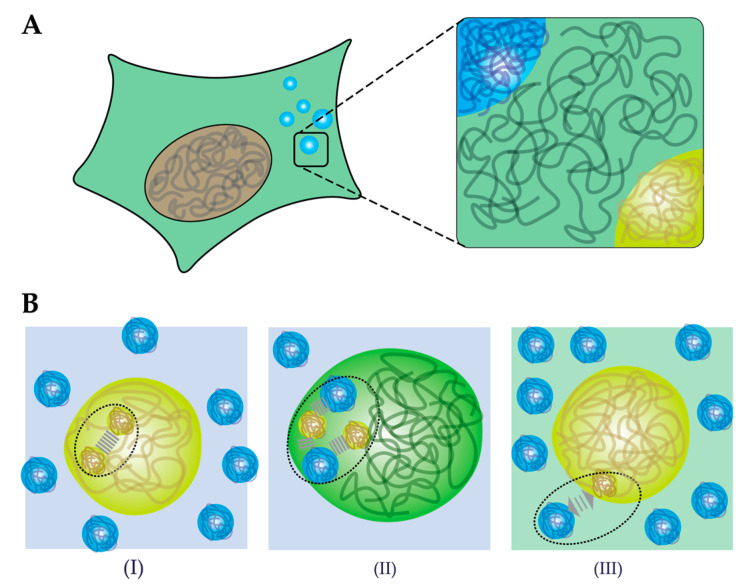
Cellular condensates might arise from both associative and segregative phase separation. (**A**) Phase separation results in a crowded protein-dense phase that is situated in the crowded cytosol (or nucleoplasm). (**B**) Three effects on phase separation caused by macromolecules: (I) LLPS is induced through depletion forces, increasing the attractive forces within the condensate. (II) LLPS is promoted through co-condensation of macromolecules through attractive interactions between the macromolecules and the proteins. (III) LLPS is promoted through segregation, as repulsive interactions between macromolecules and proteins lower the solubility of proteins.

An open question remains what the mechanism behind crowding-induced phase separation is. We propose that three effects could lead to enhanced phase separation by crowding agents (Figure 4B). First, according to classical crowding theories, volume exclusion by the crowding agents could lead to enhanced intermolecular attractions between proteins and between proteins and nucleic acids (Figure 4B,I). As a result of stronger attraction, lower concentrations of protein are sufficient to nucleate a condensed phase. Second, macromolecules could interact specifically with the proteins (Figure 4B,II). When the interactions between the “crowding” agents and biomolecules are favorable, this could enhance phase separation through co-condensation. Note that this is not a crowding effect, but rather a form of associative phase separation. Evidence from several in vitro studies indicate that even so-called inert crowding agents, like PEG and Ficoll, could be accumulated inside condensates instead of being excluded, and we will discuss this phenomenon in Section 4.2. Third, the solubility of proteins could change in the presence of crowders, which is typical of segregative phase separation (Figure 4B,III). A classical example of segregative phase separation between synthetic polymers is the combination of PEG and Ficoll [107]. Similarly, Julius et al. showed phase separation of lysozyme and PEG [101]. It is not unlikely that polymeric or protein-based crowding agents have a similar effect on the solubility of many biomolecules, in particular if they contain relatively hydrophobic sticker motifs [64]. By extension, increasing the degree of crowding in an already phase separated mixture, could lead to changes in density and fluidity of condensates, and ultimately result in aggregation. We further discuss the effect of crowding on the material properties of condensates in Section 4.3, based on several case studies.

### 4.2. Macromolecular Partioning Misleading Mechanisms of Phase Separation Regulators

As indicated, many macromolecular crowders have been shown to induce phase separation (Table 1). However, if they act as crowders to push other proteins together and facilitate phase separation, they should be excluded from the condensed phase [16]. While most studies have not looked into the localization of crowding agents, there are indications that some prototypical crowding agents do not always behave like ideal inert species that are excluded from condensed phases. In this section, we will discuss how the spatial distribution of macromolecules can reveal more about the role of macromolecules as regulators of phase separation.

#### 4.2.1. Attractive Interactions of Macromolecules can Regulate Phase Separation

Macromolecules that have been used to study crowding are normally considered neutral inert molecules [16]. Ghosh and co-workers showed that, depending on the strength of the interactions, macromolecules can either promote or inhibit phase separation [118]. Using computational models, they predicted that macromolecules with strong attractive interactions could promote phase separation [118] by co-condensation. As a model system, they used the complex phase separation of SH3_5_ with PRM_5_ proteins designed by Rosen’s lab [66,120]. Heparin, a negatively charged macromolecule that attracts the positively charged PRM_5_, promoted phase separation and became accumulated in the protein-dense phase [118]. This process is dependent on the concentration of heparin showing a reentrant behavior similar to what has been observed for RNA–protein condensates [118,121].

A second example where PEG promotes phase separation by association has been reported by the lab of Keating, who pioneered RNA-based complex coacervates as MLO model systems [122,123,124]. Marianelli and co-workers used coacervates formed from negatively charged poly-U RNA and positively charged spermine (Figure 3C), and studied the effect of macromolecular crowding on the phase transition using PEG (8 kDa) and Ficoll-70 (70 kDa) [122]. While PEG decreased the critical charge ratio between poly-U/spermine coacervates (similar to the examples in Section 4.1), this effect was negligible in the presence of Ficoll-70. Thus, the effect of PEG could not be explained by classical volume exclusion theory. Experiments with labelled Ficoll-70 and PEG revealed that only PEG was excluded from the coacervates, while Ficoll was enriched inside the dense coacervate phase [122]. Therefore, the enhanced phase separation effect of Ficoll cannot be attributed to crowding. The authors proposed that PEG (and ethylene glycol) could distort the RNA secondary structure through exclusion of water [122]. PEG destabilizes the poly-U structure, which results in a more extended U polymer, facilitating the interactions between poly-U and spermine and resulting in enhanced phase separation. Conformational changes in RNA structure have been shown to have large effects on condensate selectivity [79] and, therefore, crowding effects should be carefully considered as well.

A third type of regulator that is not directly linked to crowding is the weak interactor that inhibits phase separation of two complex-forming biomolecules. Ghosh and co-workers proposed that weak attractive interactions between the regulator and one of the phase separating molecules would result in competition between the regulator and the other phase separating molecule [118]. As an experimental example, lysozyme was used. In a previous study by Protter and co-workers, proteins such as BSA and lysozyme were shown to inhibit phase separation of hnRNPA1 [62]. This can be explained by a comparison of the partition coefficients of lysozyme and heparin for the SH3_5_/RPM_5_ system: lysozyme has a partitioning coefficient of 1, while heparin has a coefficient of 6.

#### 4.2.2. Effect of Macromolecular Size in Partitioning

Besides the type of “crowding” agent, the size of macromolecules used to promote phase separation could also impact the mechanism by which phase separation is facilitated. As an example, we want to highlight the partitioning of dextran in a study by Schuster and co-workers [68]. Schuster used condensates formed by the LAF1 RGG domain to study the partitioning of dextran of varying lengths [69]. While low molecular weight dextran (4.4 kDa) was enriched in the dense phase, partitioning decreased with increasing dextran size [68]. Dextran of 10 kDa was found to be the threshold for being equally distributed between dense and diluted phase. Dextran of high molecular weight (70 kDa) was entirely excluded from the condensates [68]. For this specific model, the effect of crowding could be studied by using dextran-70.

These examples show that macromolecules can regulate phase separation through partitioning, and that not all effects of so-called crowding agents on phase separation can be attributed to crowding. The strength of the interaction between “crowding” agents and proteins is important in these cases, just like the size of the macromolecules. These insights also have implications for phase separation inside living cells. Cells contain a large variety of small molecules, which have a weak or strong interaction with several phase separating proteins, and their partitioning behavior could be affected by crowding. As an example, Marianelli found that an oligo RNA exhibited a two-fold higher partitioning coefficient under crowded conditions [122].

### 4.3. Crowding Affects Biophysical Properties of Condensates

For many biomolecular condensates, there exists a very fine phase boundary between the liquid-to-gel (or solid) state [125]. When interactions are strengthened by the excluded volume effect and depletion, it is possible that condensates are pushed across this boundary from liquid to solid. Here, we will discuss three recently studied systems, in which the material properties of condensates have been studied under crowded conditions.

#### 4.3.1. The Effect of Crowding on FUS

Solidification of protein condensates (e.g., stress granules) has been linked to various neurodegenerative diseases such as Alzheimer’s and Parkinson’s disease, where condensates lose their liquid properties and start forming amyloid fibrils [40,100]. Molliex and Lin explained that this transition from liquid to solid state is due to stabilization of the prion-like domains (PrLD) of RNA-binding proteins, also called maturation [40,100]. As the proteins (generally FUS, but also other stress granule components) require crowders to form condensates, Kaur and co-workers studied this specific effect for FUS.

Fluorescence recovery after photobleaching (FRAP) is a technique commonly used to test whether biomolecules in the dense condensate phase are mobile or immobile [126]. The faster the recovery of the fluorescence signal after bleaching a spot, the faster the diffusion of fluorescently labelled molecules. Kaur’s study showed that full-length FUS (FUS^FL^) transits from a viscous fluid to a viscoelastic gel-like state in a crowding dependent manner [106]. This gradual effect was also observed for the FUS RGG domain, but not for the FUS prion-like domain (FUS^PrLD^). The FUS^PrLD^ showed a more switch-like behavior: above 15% PEG, no recovery was observed for FUS^PrLD^. This effect was independent of the molecular weight of PEG and dextran, and was attributed to the general increase in intermolecular interactions caused by volume exclusion.

#### 4.3.2. The Effect of Crowding on NPM1

Kriwacki’s lab observed a similar reduced mobility effect in nucleophosmin (NPM1) condensates [104,105]. Under non-crowded conditions, NPM1 requires a second macromolecule to form condensates, such as ribosomal RNA (rRNA) or proteins with an arginine-rich motif (e.g., surfeit locus protein 6 (SURF6)) [72]. When adding crowders such as Ficoll or PEG, Mitrea and co-workers observed homotypic NPM1 droplets that arise from interactions between acidic and basic tracks [105]. They then questioned how crowding influences the material properties of these NPM1–NPM1-rich phases, and by extension, the nucleolus.

Ferrolino and co-workers discovered that the mobility of proteins in the dense phase of NPM1 homotypic droplets decreased rapidly under increasing crowding conditions [103]. Where the FRAP recovery was reduced 2-fold at 5% PEG, there was almost no recovery at 15% PEG. As PEG was not accumulated in the dense phase, this effect was attributed to the excluded volume effect that compacts the disordered regions [103,127]. Maybe even more striking was the observation that NPM1 homotypic droplets do age like stress granule condensates. After 3 h, the dense phase became immobile, although this effect was reversible, in contrast to FUS ageing [40,103].

The situation was different for heterotypic droplets containing the arginine-rich protein SURF6. SURF6 accumulates in the dense NPM1 droplets and under crowded conditions, and SURF6 itself was mobile within the condensates at all tested ratios [103]. However, at low SURF6/NPM1 ratios, NPM1 behaves more like homotypic condensates. When the SURF6-to-NPM1 ratio was increased to 4, NPM1 showed a significantly higher FRAP recovery. Furthermore, Ferrolino discovered that at all ratios, the homotypic NPM1 is predominantly present at the interface, suggesting a core–shell-like structure. This could give more insight into how the fibrillar component of the nucleolus is structured.

#### 4.3.3. Crowding Has No Effect on Protein Mobility of a Synthetic Silk-Like Protein

The previous two examples of both FUS and NPM1 showing reduced mobility under crowded conditions indicate a transition from a viscous to a more gel-like state. However, within cells, many of these condensates do show liquid-like behavior and they do recover after photobleaching [8,128]. Lemetti and coworkers engineered a silk-like protein CBM–eADF3–CBM (cellulose binding modulus, CBM, linked by a silk repeat containing A- and Q-rich blocks, eADF3) that showed similar unchanged biophysical properties upon crowding [119]. While crowding with dextran did decrease the concentration required for phase separation, the FRAP recovery was not affected—almost full recovery was observed within 30 seconds. Apparently, the effective density and viscosity of the condensed phase of CBM–eADF3–CBM was not significantly affected by crowding.

## 5. Conclusions and Outlook

This review focused on the role of crowding in driving and shaping phase transitions of biomolecular components. Although major progress has been made in understanding the phenomenon of LLPS and the drivers of phase separation, the role of the dense crowded environment of the cell remains underappreciated. Most of our current understanding of the role of crowding comes from in vitro studies that aim to mimic the crowded cellular environment. The cases we have highlighted in this review show significant changes in the phase diagram, and suggest that crowding plays a very significant role in determining both the stability of mixtures of biomolecules and the extent of the region of phase separation. The addition of so-called crowding agents appears to induce or enhance LLPS in almost all in vitro studies we analyzed, but the mechanism underlying enhanced LLPS remains poorly characterized. We propose that at least three effects could lead to enhanced phase separation by crowding agents: (i) classical volume exclusion by crowding agents leads to enhanced intermolecular attractions, (ii) some macromolecules that are used as crowding agents could interact specifically with phase-separating biomolecules and enhance phase separation through co-condensation, and (iii) crowding agents could decrease the solubility of biomolecules, in a process of segregative phase separation. Moreover, these effects are likely to influence the biophysical properties of already formed condensates as well. In the complex, crowded environment of the cell, both the condensates and the crowding agents are much more diverse and heterogeneous than in any of the in vitro studies, and it is likely that all three effects play some role: weakly attractive proteins or nucleic acids could nucleate condensates, inert proteins exhibit a more idealized excluded volume effect, and weakly repulsive proteins are expected to segregate from each other. Additionally, many factors previously unaccounted for, such as chaperones, specific RNA-binding proteins, siRNAs, and the presence of lipid membranes, could also change the phase behavior of specific condensates. To fully understand each of these contributions, a more systematic approach will be needed.

## Figures and Tables

**Table 1:** Crowding agents that have been used to induce or promote protein phase separation in vitro.

Protein	Membraneless Organelle	Crowder	[Crowding] ^1^	Crowding Necessary?	Ref.
FUS	Stress Granules	PEG	2–30%	No	[39,67,99,108]
		Dextran	10%		[39,67,99]
		Ficoll-400	15%		[109]
		BSA	10%		[100]
G3BP1	Stress Granules	PEG (20k)	1–8%	Yes	[98]
		Ficoll-400	5–20%		[97]
TIA-1	Stress Granules	PEG	10%	Yes	[110]
Tau (K18)	Stress Granules	PEG	7.5%	No	[95]
Tau-187		PEG	10%	Yes	[96]
TDP-43	Stress Granules	Dextran	10%	Yes	[99,111]
hnRNPA1	Stress Granules	PEG	10–20%	No	[40,100]
		Ficoll-70	10%	No	[40,100]
		Dextran	10%		[67]
		BSA	1.5–10%		[63]
		Yeast lysate	1%		[63]
hnRNPA3	Stress Granules	Dextran	10%	No	[67]
Efhd2	Stress Granules	PEG	10%	Yes	[97]
EWSR1	Stress Granules	Dextran	10%	No	[67]
TAF15	Stress Granules	Dextran	10%	No	[67]
FMRP	Neuronal Granules	PEG	30%	No	[108]
FXR1	Neuronal Granules	BSA	3%	Yes	[112]
SPOP/cDAXC	Nuclear Speckles	Ficoll	4–10%	Yes	[113]
FBL	Nucleolus	Dextran	10%	No	[105]
NPM1	Nucleolus	PEG	5–15%	Yes ^2^	[103,104]
		Dextran	15%		[103]
		Ficoll	15%		[103]
Ddx3x	Stress Granules	PEG	10%	Yes	
LAF1-(RGG domain)	Germ Granules (P-granules)	Dextran	1%	No	[68] ^3^
FCA	(plant) nuclear bodies	PEG	10%	No	[114]
Pol-II-CTD	Transcriptional bodies	PEG	10%	Yes	[102]
		Ficoll-400	16%		[102]
NusA	Bacterial Bodies	Dextran	10%	Yes	[115]
Brd4S	Nuclear puncta	PEG	2–4%	Yes	[116]
		HeLa nuclear extract	0.3%		[116]
BuGZ	Spindle bodies	PEG	10–40%	Yes	[117]
SH3_5_/PRM_5_	Synthetic	Ficoll-70	2.5–40%	No	[118]
CBM-eADF3-CBM	Synthetic	Dextran	1–14%	No	[119]
		Ficoll	1–14%		[119]

^1^ Concentration crowding agent. ^2^ Crowding is necessary for homotypic liquid-liquid phase separation (LLPS). ^3^ Dextran was not used as a crowder, but to study partitioning of different-sized dextran polymers.

## Abbreviations

Brd4S	Bromodomain containing protein 4S
BSA	Bovine serum albumin
BuGZ	BUB3-interacting and GLEBS motif-containing protein zinc finger protein 207
CBM	Cellulose binding domain
Da	Dalton
DAXC	Death domain-associated protein-6
Ddx4	DEAD box ATPase protein 4
DNA	Deoxyribonucleic acid
dsDNA	Double stranded DNA
EFhd2	EF-hand domain-containing protein D2
EWSR1	Ewing sarcoma breakpoint region 1 protein
FBL/Fib-1	Fibulin/Fibrillarin
FCA	Flowering time control protein FCA
FMRP	Fragile X mental retardation protein
FRAP	Fluorescence recovery after photobleaching
FRET	Förster resonance energy transport
FUS	Fused in sarcoma protein
FXR1	Fragile X mental retardation syndrome-related protein 1
G3BP1	Ras GTPase-activating protein-binging protein 1
H1	Histone protein 1
hnRNPA1	Heterogenous nuclear ribonucleoprotein A1
hnRNPA3	Heterogenous nuclear ribonucleoprotein A3
IDP	Intrinsically disordered protein
IDR	Intrinsically disordered region
LAF1	Transcription factor protein LAF1
LCD	Low complexity domain
LLPS	Liquid-liquid phase separation
MLO	Membraneless organelle
NBD	Nucleic acid binding domain
Nck	Cytoplasmic protein Nck-1
NMR	Nuclear magnetic resonance
NPM1	Nucleophosmin
N-WASP	Neural Wiskott-Aldrich syndrome protein
P-body	Processing body
PEG	Polyethylene glycol
Pol-II-CTD	Polymerase-II-C-terminal domain
Poly-U	Polyuridylic acid
PrLD	Prion like domain
PRM	Proline rich motif
RGG	Arginine-Glycine-Glycine rich motif
RNA	Ribonucleic acids
RRM	RNA recognition motif
rRNA	Ribosomal RNA
SH2	Src Homology 2
SPOP	Speckle-type POZ protein
SURF6	Surfeit Locus protein 6
TAF15	TATA-binding protein-associated factor N2
TIA1	T-cell restricted intracellular antigen-1/Nucleolysin TIA1
TDP43	TAR DNA binding protein 43
ZnF	Zinc Finger domain
